# Development of a clinically practical whole-brain intracranial vessel wall MRI technique at 3 Tesla

**DOI:** 10.1186/1532-429X-18-S1-P350

**Published:** 2016-01-27

**Authors:** Zhaoyang Fan, Qi Yang, Shlee S Song, Zixin Deng, Ayesha Z Sherzai, Xiaoming Bi, Dean Sherzai, Debiao Li

**Affiliations:** 1 grid.50956.3f0000000121529905Biomedical Sciences, Cedars-Sinai Medical Center, Los Angeles, CA USA; 2 grid.50956.3f0000000121529905Neurology, Cedars-Sinai Medical Center, Los Angeles, CA USA; 3 grid.50956.3f0000000121529905Neurosurgery, Cedars-Sinai Medical Center, Los Angeles, CA USA; 4 Siemens Healthcare, Los Angeles, CA USA

## Background

T1-weighted variable-flip-angle 3D TSE has emerged as a promising intracranial vessel wall imaging technique. To increase spatial coverage and cerebrospinal fluid (CSF) attenuation, a whole-brain 0.5-mm-reoslution protocol based on an inversion-prepared 3D TSE sequence has recently been proposed at 3T. However, its 11-12-min scan time renders it clinically impractical. This work aimed to develop an expedited protocol and validate it on patients.

## Methods

Elliptical data sampling and prolonged echo train length (ETL) can be exploited to expedite the 3D TSE acquisition. However, this would reduce SNR and compromise vessel wall delineation. On the other hand, SNR is intimately related to the refocusing flip angles that are calculated for a prescribed signal evolution of a tissue with specific T1 and T2 values (denoted here as simulation T1 and T2). We hypothesized that an appropriate choice in the combination of ETL and simulation T2 may help achieve an efficient protocol.

On a 3T system, the effects of simulation T2 and ETL on wall SNR, wall-CSF CNR, and white-gray matter CNR (indicative of T1 contrast weighting) were first explored on 9 healthy subjects. Simulation T2 varied (50, 80, 110, 140, 170, 200 ms) while ETL was held fixed at 36 (5 subjects) or 60 (3 subjects), and ETL varied (36, 44, 52, 60, 68) while simulation T2 was held fixed at 170 ms (1 subject). The range of potential protocols (i.e. combinations of ETL and simulation T2) was then narrowed. Specifically, ETL = 52 combined with a simulation T2 of 140, 170, and 200 ms were respectively tested on 7 healthy subjects. In addition, a combination of ETL = 36/T2 = 100 ms was used as a reference. An optimal imaging protocol was determined from the four scans and finally applied to a pilot study comprising 18 patients with various known arterial wall disease.

## Results

**Figure 1:**
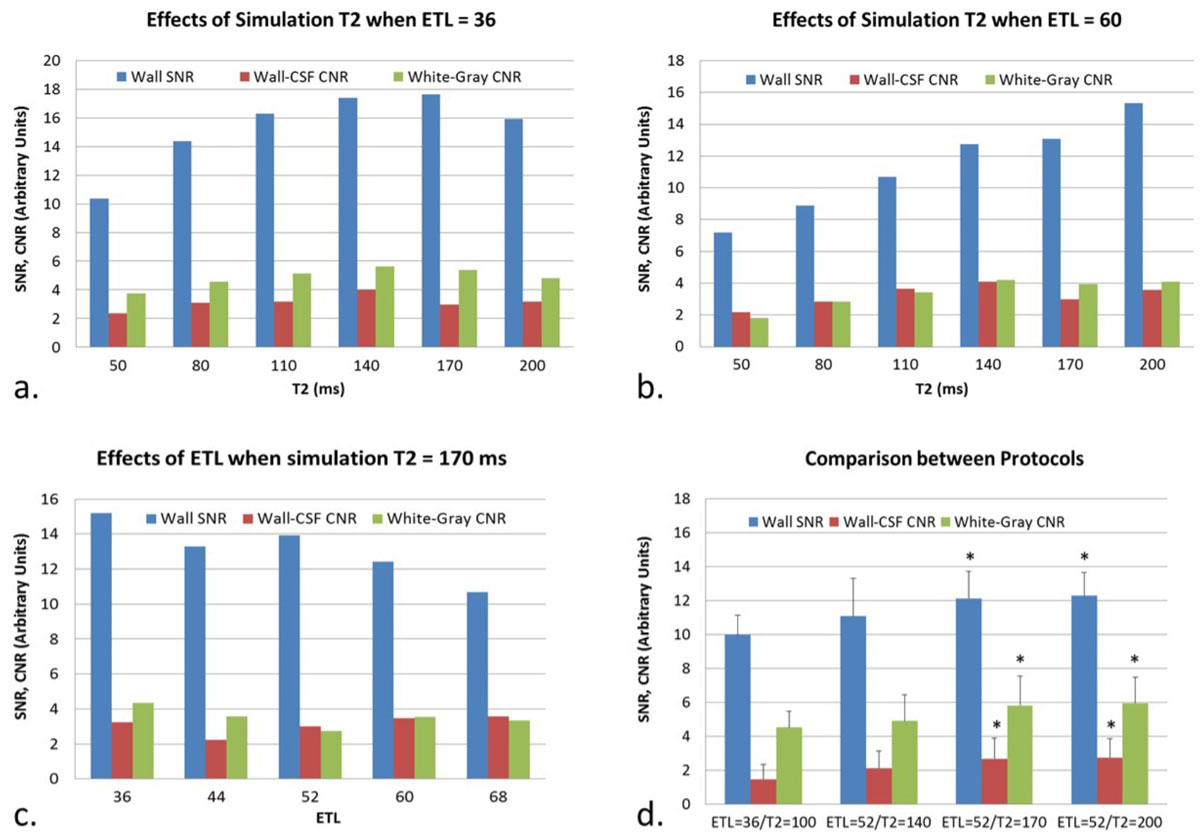
**Optimization of echo train length (ETL) and simulation T2 for an 8-min whole-brain intracranial vessel wall imaging protocol**. Increasing simulation T2 boosted SNR and CNR while ETL was fixed at 36 and 60 (a and b). As expected, SNR and CNR were reduced as ETL increased while simulation T2 was fixed (c). In a narrowed range of protocol choices (ETL = 52, T2 = 140, 170, 200 ms), an ETL of 52 in combination with a simulation T2 value of 170 ms was shown to reduce the scan time to 8 min and provide significantly increased wall SNR, wall-CSF CNR, and white-gray matter CNR, compared with the original 12 min protocol.

**Figure 2:**
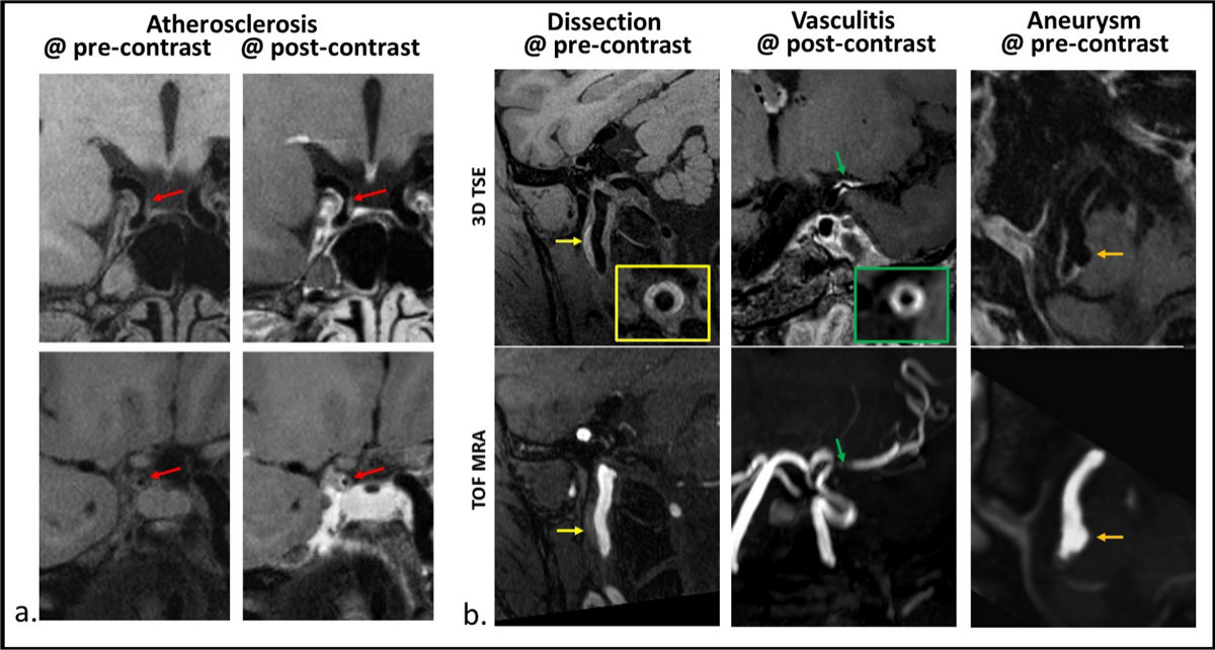
**Example clinical images obtained using the optimized whole-brain vessel wall MRI sequence (T1-weighted 3D TSE)**. The atherosclerotic plaque is depicted by 3D TSE as severe stenosis as well as eccentric wall thickening and contrast enhancement (a). Compared to TOF MRA, 3D vessel wall imaging directly depicts wall pathologies with additional information, such as hyper-intense hematoma associated with dissection and concentric contrast enhancement associated with vasculitis (b). The T1-mediated signal features within various wall pathologies on 3D TSE facilitates definitive diagnosis.

Increasing simulation T2 boosted SNR and CNR (Figure 1 a and b). As expected, SNR and CNR were reduced as ETL increased (Figure 1c). An ETL of 52 appeared to allow the scan time to reduce to 8 min while avoiding drastic SNR/CNR sacrifice. ETL = 52/T2 = 170 ms was shown to provide significantly increased wall SNR (p = 0.012), wall-CSF CNR (0.049), and white-gray matter CNR (0.019), compared with those obtained by the original 12-min protocol (Figure 1d). This combination was chosen as an optimal imaging protocol with which wall abnormalities (Figure 2) were correctly detected in all patients using their clinical diagnosis as the reference. The 8-min scan was well tolerated according to a survey. The abnormalities detected were atherosclerotic plaque in 10, vasculitis in 4, dissection in 2, aneurysm in 1, and Moyamoya disease in 1. The T1-mediated signal features within various wall pathologies facilitated definitive diagnosis.

## Conclusions

Whole-brain intracranial vessel wall evaluation at 3T is feasible within a clinically acceptable scan time - 8 min. A large-scale trial on using the technique for diagnosis of stroke etiology is underway to establish its clinical usefulness.

